# Unraveling the Role of Molecular Profiling in Predicting Treatment Response in Stage III Colorectal Cancer Patients: Insights from the IDEA International Study

**DOI:** 10.3390/cancers15194819

**Published:** 2023-09-30

**Authors:** Ippokratis Messaritakis, Eleni Psaroudaki, Konstantinos Vogiatzoglou, Maria Sfakianaki, Pantelis Topalis, Ioannis Iliopoulos, Dimitrios Mavroudis, John Tsiaoussis, Nikolaos Gouvas, Maria Tzardi, John Souglakos

**Affiliations:** 1 Laboratory of Translational Oncology, Medical School, University of Crete, 70013 Heraklion, Greece; eleni.psar2001@gmail.com (E.P.); vogiatzogloukwstas@gmail.com (K.V.); mimasf19@gmail.com (M.S.); mavroudis@uoc.gr (D.M.); johnsougl@gmail.com (J.S.); 2 Institute of Molecular Biology and Biotechnology, Foundation for Research and Technology-Hellas, 70013 Heraklion, Greece; topalis@imbb.forth.gr; 3 Laboratory of Computational Biology, Division of Basic Sciences, School of Medicine, University of Crete, 71003 Heraklion, Greece; iliop.john@gmail.com; 4 Department of Medical Oncology, University General Hospital of Heraklion, 71100 Heraklion, Greece; 5 Department of Anatomy, School of Medicine, University of Crete, 70013 Heraklion, Greece; tsiaoussis@uoc.gr; 6 Medical School, University of Cyprus, 99010 Nicosia, Cyprus; nikos.gouvas@gmail.com; 7 Laboratory of Pathology, Medical School, University of Crete, 70013 Heraklion, Greece; tzardi@med.uoc.gr

**Keywords:** colorectal cancer, stage III, molecular profiling, IDEA study, whole exome sequencing, bioinformatics

## Abstract

**Simple Summary:**

The treatment and prognosis of colorectal cancer (CRC) patients vary depending on their disease stage at diagnosis. Understanding the processes of tumorigenesis and disease development can reveal new therapeutic targets and guide patient management. On the basis of this, we investigated the molecular profiles of 237 patients with stage III CRC enrolled in the international IDEA study. We also correlated the molecular profile with Toll-like and vitamin D receptor polymorphisms, clinicopathological and epidemiological characteristics, and patient outcomes. This study suggests that the molecular characterization of tumor cells may contribute to the understanding of the biological disease course. Mutations can serve as promising prognostic biomarkers leading to better treatment options. If the results are confirmed in larger patient cohorts, then this is expected to guide clinical decision-making and personalized and improved care, and reduce treatment toxicity and patient and health system costs.

**Abstract:**

Background: This study aimed to investigate the molecular profiles of 237 stage III CRC patients from the international IDEA study. It also sought to correlate these profiles with Toll-like and vitamin D receptor polymorphisms, clinicopathological and epidemiological characteristics, and patient outcomes. Methods: Whole Exome Sequencing and PCR-RFLP on surgical specimens and blood samples, respectively, were performed to identify molecular profiling and the presence of Toll-like and vitamin D polymorphisms. Bioinformatic analysis revealed mutational status. Results: Among the enrolled patients, 63.7% were male, 66.7% had left-sided tumors, and 55.7% received CAPOX as adjuvant chemotherapy. Whole exome sequencing identified 59 mutated genes in 11 different signaling pathways from the Kyoto Encyclopedia of Genes and Genomes (KEGG) CRC panel. On average, patients had 8 mutated genes (range, 2–21 genes). Mutations in *ARAF* and *MAPK10* emerged as independent prognostic factors for reduced DFS (*p* = 0.027 and *p* < 0.001, respectively), while RAC3 and *RHOA* genes emerged as independent prognostic factors for reduced OS (*p* = 0.029 and *p* = 0.006, respectively). Right-sided tumors were also identified as independent prognostic factors for reduced DFS (*p* = 0.019) and OS (*p* = 0.043). Additionally, patients with tumors in the transverse colon had mutations in genes related to apoptosis, *PIK3*-*Akt*, *Wnt*, and *MAPK* signaling pathways. Conclusions: Molecular characterization of tumor cells can enhance our understanding of the disease course. Mutations may serve as promising prognostic biomarkers, offering improved treatment options. Confirming these findings will require larger patient cohorts and international collaborations to establish correlations between molecular profiling, clinicopathological and epidemiological characteristics and clinical outcomes.

## 1. Introduction

Colorectal cancer (CRC) is one of the most common malignancies and the second most common cause of death from cancer [1]. In 2020, 1.9 million new cases of CRC and approximately 935,000 deaths were reported [2]. By 2030, the global burden of CRC is predicted to be 60%, with more than 2.2 million new cases and 1.1 million deaths. By 2035, the total number of deaths from rectal and colon cancer is estimated to increase by 60% and 71.5%, respectively [2]. Depending on the stage of the disease at the time of diagnosis, both the treatment and prognosis differ. Patients with stage III CRC have an overall 5-year survival rate of 60%. Adjuvant chemotherapy aims to increase this rate and extend both the overall and disease-free survival [3]. Since 2004, folinic acid, fluorouracil, and oxaliplatin (FOLFOX) or capecitabine and oxaliplatin) for six months has been the standard treatment regimen [3,4]. However, oxaliplatin can lead to adverse effects, particularly peripheral sensory neuropathy [5]. 

Considering the increased incidence of the disease, toxicity of the treatment, cost, and efforts to reduce the duration of treatment for all the aforementioned reasons [5], the international IDEA study was designed to evaluate the hypothesis of non-inferiority of the 3-month vs. 6-month adjuvant chemotherapy with FOLFOX or CAPOX [6].

Although strong, AJCC/UICC-TNM staging (American Joint Committee on Cancer/Union Internationale Contre le Cancer—extent of primary tumor, regional lymph node involvement, presence of distant metastases) often fails to provide complete prognostic information because the outcome varies even among patients at the same stage [7]. Therefore, there is an urgent need to identify new tools that can contribute to CRC prognosis. The aim of the current study was to investigate the molecular profile of surgical specimens from stage III CRC patients enrolled in the international IDEA study, for whom paraffin-embedded cancer tissue was available. Following genetic profiling, correlations were performed with clinicopathological characteristics, as well as with patient outcomes. Additionally, the patients were tested for vitamin D receptor (*VDR*) and Toll-like receptor (*TLR*) gene polymorphisms in peripheral blood, as our previous studies have demonstrated the role of such polymorphisms in tumor development and progression [8,9,10].

## 2. Patients and Methods

### 2.1. Patient Enrollment

The Hellenic Oncology Research Group (HORG) enrolled 708 patients with CRC in the international IDEA study (ClinicalTrials.gov Identifier: NCT01308086). Of these, 237 stage III patients enrolled from September 2009–July 2015, with formalin-fixed paraffin-embedded (FFPE) tissues, were included in the current study (Supplementary Table S1). All patients were aged >18 years and received adjuvant chemotherapy with FOLFOX or CAPOX. None of the enrolled subjects had any other documented malignancies. 

### 2.2. Formalin-Fixed Paraffin-Embedded (FFPE) Tissues

All surgical materials were evaluated by a specialized pathologist at the Department of Pathology of the University General Hospital of Heraklion, Crete, and the most representative and enriched tumor areas were selected for dissection. Healthy tissue was used as the control tissue. The specifications of the FFPE sections used for DNA extraction were as follows: a. tissue surface area, 25 mm^2^; b. section thickness, 10 μm; c. At least 10 sections with >50% cancer cells; d. Cancer cells were collected from areas rich in cancerous tissue and avoiding healthy tissue, adipose tissue, necrotic areas, and lymphocytes that decrease the content of cancer DNA. To facilitate the collection of appropriate cells from the 10 sections, an additional section was stained with hematoxylin-eosin to locate the cancerous areas.

### 2.3. TLR and VDR Genotyping in Blood Samples

A total of 5 mL of peripheral blood in EDTA was collected from each patient, and DNA was extracted using the QIAamp DNA Blood Mini kit (QIAGEN, Hilden, Germany) according to the manufacturer’s instructions. The DNA concentration was determined using a NanoDrop ND-1000 v3.3 spectrophotometer (Thermo Fisher Scientific, Wilmington, DE, USA).

To determine the genetic variants of *TLRs* and *VDRs*, polymerase chain reaction and restriction fragment length polymorphism (PCR-RFLP) were used to determine the genetic variants of TLRs and VDRs. For *TLR2* 196-to-174 Ins/Del genetic variants, PCR was used, while *TLR4* (Asp299Gly-rs4986790 and Thr399Ile-rs4986791) and *TLR9* (T1237C-rs5743836 and T1486C-rs187084) genetic variants were determined using PCR-RFLP. The materials and conditions for each gene target have been previously described [8,9]. Similarly, for genotyping of VDR genetic variants at the *Taq*I (rs731236), *Apa*I (rs7975232), *Fok*I (rs10735810), and *Bsm*I (rs1544410) positions, PCR-RFLP was used. The reagents and PCR conditions have been previously described in detail [8,9,10]. The patients were classified as wild-type, heterozygous, or homozygous for each single nucleotide polymorphism, based on the absence or presence of the restriction site in both alleles.

### 2.4. Whole Exome Sequencing (WES)

The Illumina DNA Prep with Enrichment kit (Illumina, San Diego, CA, USA, 92122) was used for library preparation and enrichment, and sequencing of both tumor and normal tissues was conducted using the NovaSeq 6000 system (Illumina) flow cell. For each sample, 250–350 ng of high-quality DNA was quantified using a Qubit Fluorometer (Thermo Fisher Scientific, Paisley, UK), ensuring consistency across all samples. Samples with quality threshold 260/280 ratio of at least 1.8–2.2 were selected to ensure high-quality DNA and used for library preparation. Following the WES, two files (.fastq) containing sequencing data were extracted from each tissue (tumor and normal) using forward and reverse reads (2 × 150 base pairs). The coverage within the designated target region and the number of generated reads exceeded the threshold set by the manufacturer (Illumina). Specifically, the coverage reached a level of 150×, while the number of reads surpassed 40 billion.

### 2.5. Bioinformatic Analysis

**Figure 1:**
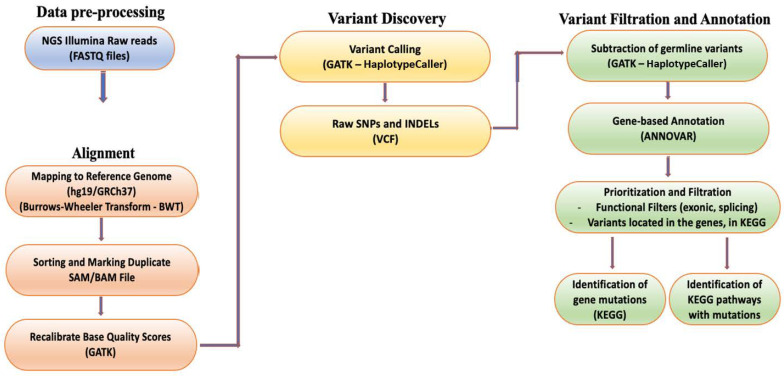
Overview of the next generation sequencing (NGS) analysis pipeline.

After obtaining the raw data from the WES analysis, a bioinformatics pipeline was used to process the data and generate interpretations. This included alignment to the human genome, variant calling, variant filtering, and annotation of the variants. The processed data were analyzed to identify somatic variants and evaluate their functional significance, particularly those associated with CRC (Figure 1) [11,12,13]. Initially, the raw sequences were aligned to the human genome (version hg19/GRCh37) [14] utilizing the Burrows–Wheeler Transform [15], and variant calling was conducted using the genome analysis toolkit (GATK, version 4.2.3) for SNPs and insertion/deletions (INDELs). This generates two variant call format files (VCF) for each patient: one for the tumor and one for the normal tissue, respectively [16]. The somatic variants were isolated by subtracting the germline variants from the tumor, and a custom gene panel was utilized based on the Kyoto Encyclopedia of Genes and Genomes (KEGG) database on CRC-correlated genes [17] (Supplementary Tables S2 and S3). Subsequently, ANNOVAR software (version 2.17) was employed to annotate the SNPs and INDELs, providing functional information that can determine the biological significance of each variant and identify CRC-associated variants [18].

To identify clinically significant variants and the signaling pathways involved, a filtering strategy was employed based on the functional position of the variants. Variants located in exons and splice sites were isolated as they are more likely to cause diseases [19]. In addition, the variants located in exons were further filtered based on their functional consequences, excluding variants causing synonymous mutations. This step considered synonymous mutations that do not affect the amino acid sequence of proteins and are therefore less likely to be clinically relevant. All analyses were run in the Anaconda Powershell Prompt (Anaconda3, Inc., Wang and Oliphant, Berlin, Germany) on Ubuntu 20.04.3 LTS. 

### 2.6. Consensus Molecular Subgroups (CMS) Assignation

Regarding the CMS subtypes, patients were classified into four subtypes according to the transcriptomics of CRC [20]. CMS1 (immune) is characterized by microsatellite instability (MSI), high levels of mutations in CpG island methylator phenotype (*CIMP*) and *BRAF* genes, and a low prevalence of *SNCA* gene mutations. It is associated with lymphocyte infiltration and immune activation, along with prominent hypermethylation and reduced signaling through the *WNT* pathway [21,22]. CMS2 (canonical) demonstrates epithelial characteristics and is marked by high chromosomal instability, an elevated count of somatic copy number alterations, as well as mutations in the *WNT* and *MYC* genes, leading to heightened activity in these intracellular signaling pathways [21,22]. CMS3 (metabolic) exhibits a distinctive global genomic and epigenomic profile with mixed features, including metabolic reprogramming and dysregulated pathways. It displays increased activity in glutaminolysis and lipidogenesis, enriched with *KRAS*-activating mutations. CMS3 presents a moderate or low mixed state of MSI, intermediate *CIMP*, and moderate activation of *WNT* and *MYC* signaling. Additionally, it is characterized by *PIK3CA* mutations and *IGBP3* overexpression but lacks *BRAF* mutations [21,22]. CMS4 (mesenchymal) is typified by positive gene regulation and the overexpression of proteins involved in stromal infiltration, mesenchymal activation, extracellular matrix remodeling, neoangiogenesis, prominent TGF-β activation, and complement pathways. The presence of *CTNNB1* mutations is a distinguishing feature of CMS4 [22].

### 2.7. Statistical Analysis

After characterizing the patients’ molecular profiles, the clinical, pathological, and epidemiological characteristics were examined to determine their association with patient outcomes. Disease-free survival (DFS) and overall survival (OS) were calculated from the day of tumor excision until the first documented recurrence or death, respectively. Recurrence was defined as the presence of metastatic disease, local recurrence, or a second primary tumor. The possible associations between baseline characteristics, recurrence, and individual or concurrent mutations were compared using the 2-sided Fisher exact test for categorical variables. The association between risk factors and time-to-event endpoints was evaluated using the log–rank test, and the Kaplan–Meier method was used to generate DFS and OS curves. Univariate and multivariate Cox regression analyses were conducted to evaluate the correlation between the potential prognostic factors and DFS or OS. Statistical significance was defined as *p* ≤ 0.05, and the statistical tool used was SPSS v. 26. 

Additionally, Monte Carlo simulation methods were performed. The Monte Carlo approach, named after the Monte Carlo Casino in Monaco due to its reliance on stochastic principles, is a computational procedure that employs random sampling to tackle intricate mathematical and statistical quandaries. This method entails the generation of random samples from known probability distributions, subsequently utilizing these samples to approximate solutions to problems that defy analytical resolution [23,24,25]. The results indicate a high level of confidence (99%), at the 95% significance level, with the predicted statistical power deviating by no more than 4% from the values generated by simulation across various model parameters. This equates to sample size discrepancies of fewer than four subjects and discrepancies in the detectable accuracy difference of less than 0.6%.

## 3. Results

### 3.1. Patients

The current investigation enrolled 237 patients with stage III CRC, and their characteristics are displayed in Table 1 and Supplementary Table S1. Among these patients, 151 (63.7%) were male, 159 (67.1%) were <70 years old (median: 64 years, range: 18–84), 158 (66.7%) had tumor localization in the left colon, and 132 (55.7%) patients underwent CAPOX as adjuvant chemotherapy. Of the entire patient population, 116 (48.9%) were administered a 3-month treatment regimen, while 121 (51.1%) were administered a 6-month treatment regimen. 

### 3.2. TLR and VDR Analysis

For *VDR* and *TLR* gene polymorphisms, 84 patients were analyzed according to sample availability. Regarding *VDRs*, 10 (11.9%), 7 (8.2%), 5 (6.0%), and 17 (20.2%) patients presented *Taq*I, *Apa*I, *Fok*I, and *Bsm*I homozygous phenotypes, respectively (Table 2). Moreover, regarding *TLRs*, 48 (57.1%), 38 (46.4%), 38 (46.4%), 37 (44.0%), and 37 (44.0%) patients presented *TLR2* 196-to-174, *TLR4*-Asp299Gly, *TLR4*-Thr399Ile), *TLR9*-T1237C, and *TLR9*-T1486C homozygous phenotype, respectively (Table 2). Patients with TaqI, ApaI, and BsmI wild-type alleles are more likely to survive (*p* = 0.005; *p* = 0.021 and *p* = 0.033, respectively).

### 3.3. Annotated Variants for Each Position

The KEGG CRC panel detected 85,871 uniquely annotated variants. Of these, 602 were detected in exonic positions, including 25 splice variants. The remaining variants were non-coding, with 81,555 intronic variants being the most common, followed by 2184 UTR variants, 788 downstream variants, and 742 upstream variants.

### 3.4. Identification of Mutated Genes

**Figure 2:**
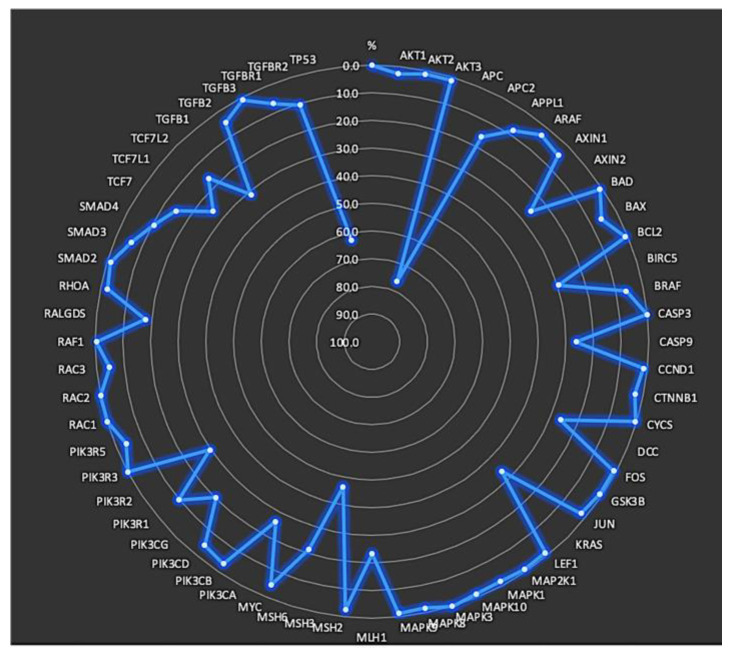
Frequency of mutations in each gene located in exons and splicing sites.

**Figure 3:**
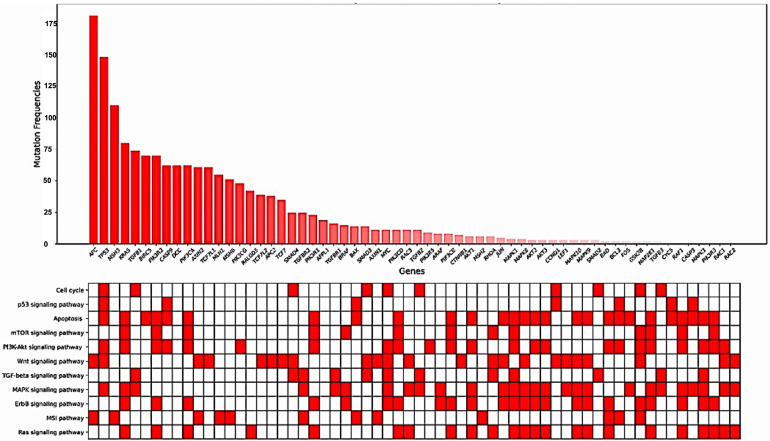
Gene mutation frequencies with KEGG pathway annotations.

Mutated genes within the KEGG CRC gene panel for each patient were identified. On average, the patients exhibited eight mutated genes (range, 2–21 genes). The frequencies of mutations in each gene, specifically in exons and splicing sites, are presented in Table 3 and Figure 2 and Figure 3.

From this analysis, it was observed that certain groups of patients had a higher mutation frequency in specific genes (Table 4). In brief, males had a significantly higher frequency of mutations in the *JUN* and *MAPK3* genes than females (*p* = 0.05, *p* = 0.05, respectively). In terms of age groups, patients below 70 years of age had a higher frequency of mutations in *TGFBR1* than those ≥70 years of age (*p* = 0.012). Similarly, patients 51–70 years old had more frequent mutations in *BAD* (*p* < 0.001), *RAC* (*p* = 0.016), *AKT*, *AKT2*, *AKT3*, *APC*, *APPL1*, *AXIN1*, *AXIN2*, *BIRC5*, *DCC*, *GSK3B*, *KRAS*, *MAPK1*, *MAPK8, MAPK9*, *MAPK10*, *MLH1*, *MSH6*, *PΙK3CA*, *PIK3R1*, *PIK3R2*, *PIK3R5*, *RAF1*, *RALGDS*, *SMAD2*, *SMAD3*, *TCF7L2*, *TGFB1,* and *TGFB2* genes (*p* = 0.037). Moreover, mutation rates in *ARAF*, *MAPK10*, *CASP*, *TCF7*, and *TGFB3* genes were significantly higher in patients relapsed after adjuvant treatment (*p* = 0.027, *p* = 0.044, *p* = 0.003, and *p* = 0.037, respectively).

Subsequently, it was demonstrated that patients with tumors located in the transverse colon, homozygous for mutated *VDR* alleles *(TaqI*, *ApaI*, *FokI*, *BsmI)* and homozygous for mutated *TLR9* alleles (T1237C and T1486C) had mutations in genes that are mainly involved in the apoptosis, *PIK3-AKT*, *Wnt,* and *MAPK* signaling pathways (Table 5).

### 3.5. Clinical Outcome Based on Molecular Profile and Patients’ Characteristics

**Figure 4:**
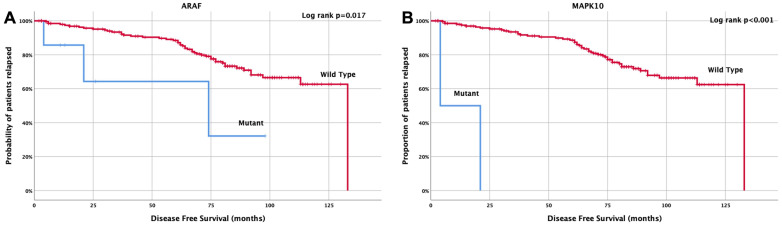
Kaplan Meier curve for disease-free survival (DFS) according to (**A**) *ARAF* and (**B**) *MAPK10* mutations.

Regarding disease-free survival (DFS), it was demonstrated that patients with *ARAF* mutations had a significantly shorter DFS (74 months, 95% CI: 29.3–154.9 months) compared with those with wild-type ARAF mutations (133 months, 95% CI: 101–138 months, *p* = 0.017) (Figure 4A). Similarly, patients with *MAPK10* mutations exhibited a significantly shorter DFS compared with wild-type patients (12.5 months, 95% CI: 0.0–29 months vs. 108 months, 95% CI: 101–114 months; *p* < 0.001) (Figure 4B).

**Figure 5:**
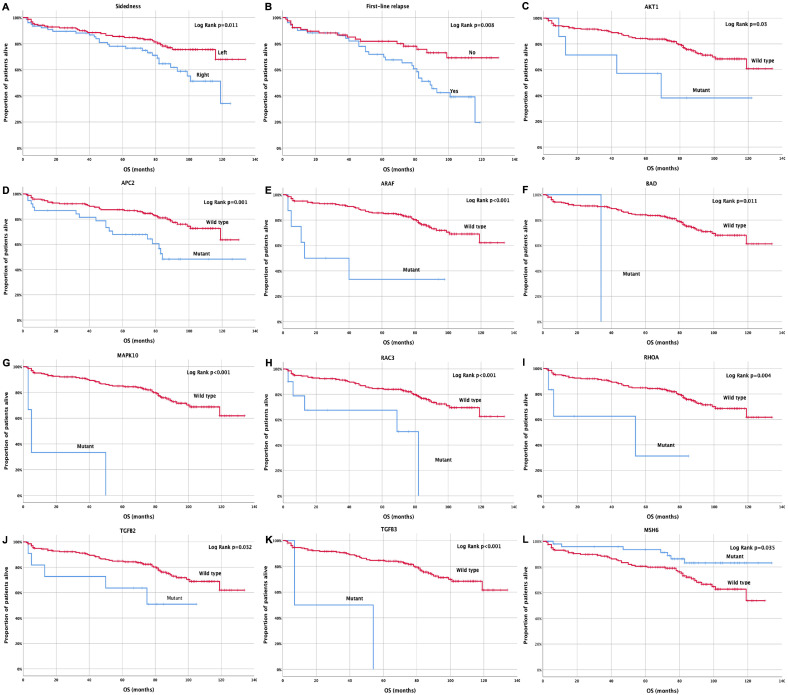
Kaplan Meier curve for overall survival (OS) according to (**A**) tumor sidedness, (**B**) relapse status, (**C**–**L**) *AKT1*, *APC2*, *ARAF*, *BAD*, *MAPK10*, *RAC3*, *RHOA*, *TGFB2*, *TGFB3,* and *MSH6* mutations.

Similarly, it was demonstrated that patients with right-sided tumors experienced a significantly shorter overall survival (OS) compared with patients with left-sided tumors (92.1 months, 95% CI: 82.5–122 months vs. 111.3 months, 95% CI: 104–135 months; *p* = 0.011) (Figure 5A). Moreover, relapsed patients demonstrated a significantly shorter OS compared with those non-relapsed (81.1 months, 95% CI: 70.4–118 months vs. 104.3 months, 95% CI: 93.9–130 months; *p* = 0.008) (Figure 5Β). Moreover, patients with *AKT1*, *APC2*, *ARAF*, *BAD, MAPK10, RAC3, RHOA, TGFB2,* and *TGFB3* mutations exhibited a significantly shorter OS compared to wild-type patients (68.9 vs. 107.9 months, *p* = 0.03; 89.7 vs. 109.4 months, *p* = 0.001; 43.3 vs. 109.4 months, *p* < 0.001; 34 vs. 107.7 months, *p* = 0.011; 19.3 vs. 108.6 months, *p* > 0.001; 55.6 vs. 109 months, *p* < 0.001; 45.2 vs. 108.5 months, *p* = 0.004; 69.5 vs. 109.7 months, *p* = 0.032; 30.5 vs. 108.1 months, *p* < 0.001, respectively) (Figure 5C–K). Finally, patients with *MSH6* gene mutations had a significantly longer OS compared with wild-type patients (120.7 months, 95% CI: 111.3–130.1 months vs. 100.9 months, 95% CI: 93.7–108.1 108.1 months; *p* = 0.008) (Figure 5L). Moreover, regarding the CMS subtypes, 5.5% of the patients were classified as CMS1 subtype based on *BRAF* and *MSI* pathway mutations; 2.5% of the patients were classified as CMS2, based on *Myc* and *Wnt* pathway mutations; 32.9% of the patients were characterized as CMS3, based on *KRAS* and *PIK3CA* mutations; and 3.0% of the patients were classified as CMS4, based on *CTNNB1* mutations. Some patients presented mixed subtypes (CMS1/CMS2: 0.4%; CMS1/CMS3: 0.4%; CMS2/CMS3: 1.7%) while 53% of these patients were not categorized in any of the above subtypes, and this might be due to mixed subtypes with intratumoral heterogeneity [21,22] (Supplementary Table S1).

### 3.6. Univariate and Multivariate Cox-Regression Analysis

Univariate analysis revealed that tumor localization in the right colon and *ARAF* and *MAPK10* mutations were associated with reduced DFS (Table 6). Multivariate analysis confirmed that tumor localization and *ARAF* and *MAPK10* mutations were independent predictive factors of reduced DFS (HR = 2.1; 95% CI: 1.1–4.0; *p* = 0.019; HR = 3.9; 95% CI: 1.2–13.1; *p* = 0.027; HR = 49; 95% CI: 9.8–244.1; *p* < 0.001) (Table 6).

Similarly, right-sided tumors and *AKT1, APC2, ARAF, BAD, MAPK10, RAC3, RHOA, TGFB2*, and *TGFB3* were associated with an increased risk of shorter OS. In contrast, *MSH6* mutations were demonstrated to be a good prognostic factor, as they were associated with a reduced risk for shorter OS (Table 6). Moreover, in order to confirm the significant relationship of the aforementioned mutations with patients’ survival, a resampling was performed using the Monte Carlo methodology (Confidence level: 99%). Based on the Monte Carlo method, a significant relationship was observed between most of these mutations and patients’ survival (Table 6). In brief, sidedness and eight genes (*AKT1*, *APC2*, *ARAF*, *MAPK10*, *MSH6*, *RAC3,* and *TGFB3*) demonstrated statistical significance with 0.799 ± 0.028 sensitivity ± standard deviation and 0.711 ± 0.087 sensitivity ± standard deviation (Table 6). Multivariate analysis revealed that right-sided tumors and *RAC3* and *RHOA* gene mutations emerged as independent predictors of reduced OS (HR = 2.2; 95% CI: 1.0–4.5; *p* = 0.043; HR = 3.5; 95% CI: 1.1–10.7; *p* = 0.029 and HR = 9.5; 95% CI: 1.9–47.7; *p* = 0.006) (Table 6).

## 4. Discussion

Despite the potential benefits of presymptomatic screening and available treatments, CRC continues to be a significant public health concern [26]. Understanding the processes involved in CRC development and progression can help to identify new targets for treatment. Structural and functional changes in the DNA can offer vital insights into patient management [27,28]. As the normal colonic epithelium transforms into cancerous tissue, various mutations occur, leading to adenoma formation [29,30,31,32,33,34,35,36]. Extensive cancer cell proliferation through the *RAS-RAF-MEK-ERK* signaling pathway drives carcinogenesis, tumor invasion, and metastasis [37]. Moreover, the immune responses to cancer cells differ among patients with mutations [38,39,40,41,42,43,44].

The objective of this study was to analyze genetic changes in surgical samples from patients with stage III CRC and detect *VDR* and *TLR* gene polymorphisms in peripheral blood samples. The study included 237 patients, 84 of whom had available blood samples. The WES and KEGG gene panel for CRC revealed 59 mutated genes belonging to 11 distinct signaling pathways. Of these, mutations in *APC2*, *BRAF*, *MAPK10*, *MLH1*, *MSH6*, *RHOA*, *TGFB*, and *TGFB2* have been linked to a significant impact on patient survival. *APC*, *TP53*, *KRAS*, and *MSH3* were the most commonly observed mutations in this study.

APC encodes an anti-tumor protein that competes with the Wnt signaling pathway and is involved in cell migration, adhesion, and apoptosis. *APC* mutations are responsible for familial adenomatous polyposis (FAP), an autosomal dominant precancerous disease that typically leads to malignancy. *APC* mutations are commonly observed in CRC cases [45]. Similarly, *APC2* mutations, which are directly associated with *APC*’s tumor-suppressive function [46], have been linked to worse prognosis in CRC patients [47,48]. This study confirms that *APC2* mutations in patients with stage III CRC are associated with lower overall survival but do not represent an independent prognostic factor. *TP53* encodes an anti-tumor protein that regulates the expression of target genes, leading to cell cycle arrest, apoptosis, senescence, DNA repair, or metabolic changes. Similarly to *APC*, *TP53* are frequently observed in CRC cases [45]. Furthermore, mutations in the *APC2* gene, which are directly linked to *APC*’s tumor-suppressive function [46], are also associated with worse prognosis in CRC patients [47,48]. This study confirms that while *APC2* mutations in stage III CRC patients are linked to lower overall survival, they do not represent an independent prognostic factor. Various human cancers, including approximately 60% of CRC, are associated with mutations in the *TP53* gene [49,50]. Prior studies have shown that mutations in *TP53* resulting in the loss of its transcriptional activity can lead to uncontrolled cellular proliferation in multiple organs, including the colon [51]. Similarly, *KRAS* mutations are the primary indicators of gastrointestinal cancers and are found in approximately 40% of patients with CRC (stage II-IV) [52]. They serve as negative prognostic factors for carcinogenesis and anti-*EGFR* therapy [53] because intracellular signal interruption leads to uncontrolled cellular proliferation and cancer. *MSH3* mutations have been mainly linked to endometrial cancer, but there are reports of its relationship with inflammatory processes, such as ulcerative colitis and Crohn’s disease, which considerably increase the likelihood of CRC development [54,55]. *MSH3*-associated CRC seems to follow the classic *APC* pathway, as patients with adenomas and CRC carrying *APC* mutations showed *MSH3* deficiency [56], as confirmed in this study. In addition to the common mutations detected in the patient group, mutations in *AKT1*, *ARAF*, *BAD*, *MAPK10*, *RAC3*, *RHOA*, *TGFB2*, and *TGFB3* were associated with worse prognosis in this study. Furthermore, mutations in *ARAF* and *MAPK10* were identified as independent prognostic factors for DFS, whereas mutations in *RAC3* and *RHOA* were identified as independent prognostic factors for decreased OS. 

Regarding the detection of gene polymorphisms in the blood, it was demonstrated that patients with VDR gene polymorphisms have shorter survival rates, and this is in agreement with our previous demonstrations in various cohorts [8,9,10].

This study sheds light on the association between mutations in genes involved in signaling pathways such as *PI3K-Akt*, *MAPK*, apoptosis, and CRC. To the best of our knowledge, this is the first report of its kind in the literature. The reactivation of embryonic self-renewal pathways, such as Hedgehog, Notch, and *TGFB/Stat3*, is characteristic of most tumors, including CRC. The *Wnt* pathway is also essential in most CRC. Targeting embryonic pathways directly is likely to be more effective against stem and differentiated cancer cells [57,58,59]. Tumors that are addicted to increased regulated activity of the embryonic pathway, in combination with high tumor heterogeneity, may be more vulnerable to such therapies [60,61,62]. Patients with *VDR* polymorphisms had concurrent mutations in genes involved in cell cycle, apoptosis, *PI3K-Akt*, *WNT*, *MAPK*, *ErbB*, MSI, and *RAS*. Similarly, *TLR9* polymorphisms were associated with mutations in genes involved in apoptotic signaling pathways, *PI3K-Akt*, and *Wnt*. Previous studies from our group have demonstrated that higher detection of *TLR* and *VDR* polymorphisms in CRC patients, especially advanced-stage patients, highlights the role of these polymorphisms in carcinogenesis, disease progression, and ultimately, patient survival [9,10,63]. Regarding DFS, tumors in the sigmoid or right colon and mutations in the *ARAF* and/or *MAPK10* genes were associated with shorter DFS, a fact that has been confirmed in previous studies [64,65,66]. To our knowledge, this is the first study to highlight the role of *ARAF* and *MAPK10* mutations as independent prognostic factors for decreased DFS.

The observation of statistically lower OS in patients with right colon tumors and gene mutations has been confirmed in the literature. Borakati et al. conducted a retrospective study and found that tumors in the right colon were independent prognostic factors for reduced OS after hepatic metastasectomy, regardless of the higher rates of liver metastases and larger metastases in the left colon [67]. Patients with mutations in genes, such as *AKT1*, *APC2*, *ARAF*, *BAD*, *MAPK10*, *RAC3*, *RHOA*, *TGFB2*, and *TGFB3* had significantly reduced OS, as reported in other studies that also linked *APC2*, *RHOA*, and *TGFB* mutations to worse prognosis [47,48,68]. Conversely, studies have shown that mutations in *MSH6* are associated with a lower risk of developing CRC, and patients with such mutations have a milder clinical presentation [69,70]. In the present study, patients with *MSH6* mutations had a significantly longer OS, confirming that *MSH6* mutations are good prognostic factors. Moreover, regarding the CMS subtypes, in the current study, it was demonstrated that the patients were classified as CMS1 subtype based on *BRAF* and *MSI* pathway mutations. Such a subtype may lead to worse survival rates after relapse, as compared to CMS2 subtype, which has been classified based on *Myc* and *Wnt* pathway mutations. CMS3 and CMS4 have also been associated with bad survival rates [21,22].

Furthermore, whether the mutations related to patients’ survival were due to chance was assessed via Monte Carlo simulations. Sidedness and eight mutations were associated with patients’ survival, which confirms the univariate analysis results.

To determine whether the results of the present work could be further validated by other studies, we examined the available data on the National Cancer Institute’s GDC data portal [71]. Case filters specifying the location of the primary tumor and clinical filters specifying gender, age, and location were applied, and the information regarding the genes of the study and the corresponding survival plots were analyzed. Consistent with the result of the current study, patients with tumors in the colon had mutations in the *KRAS* gene more frequently than patients with tumors in the sigmoid (*p* < 0.001). Moreover, patients with *AKT1, ARAF,* and *RHOA* mutations exhibited a significantly shorter OS compared to wild-type patients (*p* < 0.001, *p* < 0.001, *p* < 0.001, and *p* < 0.001, respectively) [72]. All of the above are in agreement with our results. Moreover, *MSH6* mutations are significantly correlated with previous studies, as demonstrated on the National Cancer Institute’s GDC data portal [73], and this is also in agreement with our results [71]. Concurrent mutations (co-mutations) are a significant factor that have been minimally investigated in CRC. Studies in patients with non-small cell lung cancer have shown distinct biological behavior and prognosis in *KRAS/LKB1*, *KRAS/TP53*, or *KRAS/p16* mutated tumors [74]. Additionally, our group has previously reported the importance of evaluating the loss of *LKB1* through immunohistochemistry in early-stage CRC, particularly in *BRAF*^V600E^ mutated tumors [75]. In the present study, several concurrent mutations were detected in patients, but no correlation was found with clinical/pathological characteristics or patient prognosis.

## 5. Conclusions 

In conclusion, molecular characterization of cancer cells can enhance our understanding of the biological progression of this disease [76,77]. The findings of this study suggest that mutations are promising prognostic biomarkers. As personalized medicine has become the primary mode of therapy, knowledge of the precise mutation status of patients with CRC can lead to better therapeutic choices. However, further research is necessary with a larger patient cohort and international collaborations to confirm the correlation between patients’ molecular profiles, clinicopathological and epidemiological characteristics, and outcomes. Such research is expected to contribute to more precise clinical decision-making, personalized and improved care, and reduced toxicity of treatment, costs to patients, and burden on health systems.

## Figures and Tables

**Table 1:** Patient characteristics.

Characteristics	Number of Patients (n = 237)	%
Median age (range)	64 (18–84)	
<70	159	67.1
≥70	78	32.9
Gender		
Males	151	63.7
Females	86	36.3
Tumor location		
Cecum	38	16.0
Ascending	42	17.7
Transverse	22	9.3
Descending	24	10.1
Sigmoid	111	46.8
Sidedness		
Left	158	66.7
Right	79	33.3
Performance status		
0–1	236	99.6
>2	1	0.4
Regimen		
Folfox	105	44.3
Capox	132	55.7
Treatment duration		
3 months	116	48.9
6 months	121	51.1

**Table 2:** *VDR* and *TLR* gene polymorphism detection.

Polymorphism	Gene Target	Detection	No of Patients (N-84)	%	Relapse (*p-*Value)	Better Survival (*p-*Value)
*VDR*	*Taq*I	Homozygous	10	11.9	0.185	0.05
		Heterozygous	36	42.9		
		Wild-type	38	45.2		
	*Apa*I	Homozygous	7	8.3	0.144	0.021
		Heterozygous	28	33.3		
		Wild-type	49	58.3		
	*Fok*I	Homozygous	5	6	0.133	0.104
		Heterozygous	39	46.4		
		Wild-type	40	47.6		
	*Bsm*I	Homozygous	17	20.2	0.220	0.033
		Heterozygous	51	60.7		
		Wild-type	16	19		
*TLR*	*TLR2* 196-to-174	Homozygous	48	57.1	0.126	0.180
		Heterozygous	36	42.9		
		Wild-type	-	-		
	*TLR4*-Asp299Gly	Homozygous	39	46.4	0.254	0.115
		Heterozygous	43	51.2		
		Wild-type	2	2.4		
	*TLR4*-Thr399Ile	Homozygous	39	46.4	0.254	0.115
		Heterozygous	43	51.2		
		Wild-type	2	2.4		
	*TLR9*-T1237C	Homozygous	37	44	0.114	0.185
		Heterozygous	47	56		
		Wild-type	-	-		
	*TLR9*-T1486C	Homozygous	37	44	0.114	0.185
		Heterozygous	47	56		
		Wild-type	-	-		

**Table 3:** Frequency of mutated patients in each gene.

Gene	Mutant Patients
AKT1	6
AKT2	3
AKT3	3
APC	181
APC2	38
APPL1	19
ARAF	8
AXIN1	11
AXIN2	61
BAD	2
BAX	14
BCL2	2
BIRC5	70
BRAF	15
CASP3	0
CASP9	62
CCND1	3
CTNNB1	7
CYCS	1
DCC	62
FOS	2
GSK3B	2
JUN	5
KRAS	80
LEF1	3
MAP2K1	2
MAPK1	4
MAPK10	3
MAPK3	0
MAPK8	4
MAPK9	3
MLH1	55
MSH2	6
MSH3	110
MSH6	51
MYC	11
PIK3CA	62
PIK3CB	8
PIK3CD	11
PIK3CG	48
PIK3R1	23
PIK3R2	70
PIK3R3	0
PIK3R5	9
RAC1	0
RAC2	0
RAC3	11
RAF1	1
RALGDS	42
RHOA	6
SMAD2	3
SMAD3	14
SMAD4	25
TCF7	35
TCF7L1	61
TCF7L2	39
TGFB1	74
TGFB2	11
TGFB3	2
TGFBR1	16
TGFBR2	25
TP53	148

**Table 4:** Frequency of mutations according to patients’ characteristics.

Characteristics	Gene	No % (*p* Value)
**Gender**		
**Male vs. Female**	*JUN*	43.4% vs. 25.0% (0.05)
	*MAPK3*	57.5% vs. 31.1% (0.05)
**Sideness**		
**Left vs. Right**	*MLH1*	80% vs. 20% (0.007)
	*MSH6*	84.3% vs. 15.7% (0.001)
	*TCF7L1*	77% vs. 33% (0.019)
**Location**		
**Colon vs. Sigmoid**	*DCC*	90.3% vs. 9.7% (0.04)
	*KRAS*	88.8% vs. 11.2% (0.048)
	*TGFBR2*	96% vs. 4% (0.043)
**Age**		
**<70 vs. ≥70**	*TGFBR1*	57.5% vs. 33% (0.012)
**51–70 vs. ≥70 vs. <50**	*BAD*	56.1% vs. 30.7% vs. 12.7% (<0.001)
**51–70 vs. ≥70 vs. <50**	*RAC*	56.1% vs. 30.7% vs. 12.7% (0.016)
**51–70 vs. ≥70 vs. <50**	*AKT*, *AKT2*, *AKT3*, *APC*, *APPL1*, *AXIN1*, *AXIN2*, *BIRC5*, *DCC*, *GSK3B*, *KRAS*, *MAPK1*, *MAPK8*, *MAPK9*, *MAPK10*, *MLH1*, *MSH6*, *PIK3CA*, *PIK3R1*, *PIK3R2*, *PIK3R3*, *PIK3R5*, *RAF1*, *RALGD5*, *SMAD2*, *SMAD3*, *TCF7L2, TGFB1*, *TGFB2*	56.1% vs. 30.7% vs. 12.7% (0.037)
**Relapse post Adj Chemotherapy**		
**Yes vs. No**	*ARAF, MAPK10*	71.7% vs. 20.5% (0.027)
	*CASP3*	75.5% vs. 22.6% (0.044)
	*TCF7*	75% vs. 21.2% (0.003)
	*TGFB3*	74.5% vs. 22.2% (0.037)

**Table 5:** Correlation of mutated signaling pathways and patients characteristics (X=where correlation was observed.

	Cell Cycle	Apoptosis	PI3K-Akt	Wnt	MAPK	ErbB	MSI	RAS
Transverse Colon		X	X	X	X			
TaqI Homozygous	X	X	X	X		X	X	X
ApaI Homozygous		X	X	X	X			
FokI Homozygous		X	X	X				
BsmI Homozygous		X	X	X				
TLR9-T1237C Homozygous		X	X	X				
TLR9-T1486C Homozygous		X	X	X				

**Table 6:** Univariate and multivariate Cox regression analysis.

Feature	Univariate				Monte Carlo			Multivariate			
	DFS		OS		OS			DFS		OS	
Feature	HR (95%CI)	*p*-Value	HR (95%CI)	*p*-Value	*p-*Value	Sensitivity	Specificity	HR (95%CI)	*p*-Value	HR (95%CI)	*p*-Value
Sidedness (Right vs. Left)	1.9 (1.2–3.2)	0.012	2.1 (1.0–4.0)	0.043	0.004	0.822	0.635	2.1 (1.1–4.0)	0.019	2.2 (1.0–4.5)	0.043
*AKT1* (Mutant vs. Wild-type)			2.9 (1.1–8.2)	0.039	0.048	0.771	0.721				
*APC2* (Mutant vs. Wild-type)			2.5 (1.4–4.5)	0.002	0.004	0.806	0.776				
*ARAF* (Mutant vs. Wild-type)	3.8 (1.2–12.1)	0.027	5.0 (2.0–12.7)	0.001	0.026	0.804	0.682	3.9 (1.2–13.1)	0.027		
*BAD* (Mutant vs. Wild-type)			8.6 (1.1–64.4)	0.036	0.389	0.773	0.759				
*MAPK10* (Mutant vs. Wild-type)	43.0 (9.1–203.7)	<0.001	15.1 (4.6–50.3)	<0.001	0.017	0.818	0.654	49.0 (9.8–244.1)	<0.001		
MSH6 (Wild-type vs. Mutant)			2.3 (1.0–5.1)	0.041	0.041	0.775	0.756				
*RAC3* (Mutant vs. Wild-type)			4.7 (1.8–11.9)	0.001	0.031	0.794	0.685			3.5 (1.1–10.7)	0.029
*RHOA* (Mutant vs. Wild-type)			4.8 (1.5–15.6)	0.009	0.145	0.823	0.738			9.5 (1.9–47.7)	0.006
*TGFB2* (Mutant vs. Wild-type)			2.6 (1.1–6.7)	0.040	0.091	0.792	0.717				
*TGFB3* (Mutant vs. Wild-type)			9.0 (2.1–37.8)	0.003	0.048	0.800	0.804				

## Data Availability

All relevant data are within the paper and its Supplementary Information files. Supplementary Table S1: Raw patient data.

## Supplementary Materials

The following supporting information can be downloaded at: https://www.mdpi.com/article/10.3390/cancers15194819/s1, Table S1: Raw patient data; Table S2: Gene panel for colorectal cancer (CRC) based on Kyoto Encyclopedia of Genes and Genomes (KEGG); Table S3: Signaling pathways and the associated genes for colorectal cancer (CRC) based on Kyoto Encyclopedia of Genes and Genomes (KEGG); Table S4: Detection of genes and position in chromosomes for tumor samples.
