# Supplementary material for: Unraveling the Role of Molecular Profiling in Predicting Treatment Response in Stage III Colorectal Cancer Patients: Insights from the IDEA International Study
Source: Cancers (Basel). 2023 Sep 30;15(19):4819. doi: 10.3390/cancers15194819 (PMC10571744; doi:10.3390/cancers15194819)
Supplement: Supplementary file 1 [file cancers-15-04819-s001.zip › Supplementary Table S2.pdf]

Supplementary Table S2. Gene panel for colorectal cancer (CRC) based on Kyoto Encyclopedia of Genes and Genomes (KEGG).

Gene	Chromosome	Location Based on GRCh37.p13 Assembly		Source [https://www.ensembl.org/index.html]
		Start	End	
AKT1	14	105235686	105262085	Ensembl:ENSG00000142208
AKT2	19	40736224	40791252	Ensembl:ENSG00000105221
AKT3	1	243651535	244014381	Ensembl:ENSG00000117020
APC	5	112043195	112181936	Ensembl:ENSG00000134982
APC2	19	1450120	1473243	Ensembl:ENSG00000115266
APPL1	3	57261757	57307499	Ensembl:ENSG00000157500
ARAF	X	47420604	47431307	Ensembl:ENSG00000078061
AXIN1	16	337440	402723	Ensembl:ENSG00000103126
AXIN2	17	63524681	63557766	Ensembl:ENSG00000168646
BAD	11	64037300	64052176	Ensembl:ENSG00000002330
BAX	19	49458132	49465055	Ensembl:ENSG00000087088
BCL2	18	60790579	60987002	Ensembl:ENSG00000171791
BIRC5	17	76210334	76221716	Ensembl:ENSG00000089685
BRAF	7	140413128	140624729	Ensembl:ENSG00000157764
CASP3	4	185548850	185570601	Ensembl:ENSG00000164305
CASP9	1	15817896	15851285	Ensembl:ENSG00000132906
CCND1	11	69455924	69469242	Ensembl:ENSG00000110092
CTNNB1	3	41240996	41281934	Ensembl:ENSG00000168036
CYCS	7	25158275	25164879	Ensembl:ENSG00000172115
DCC	18	49866567	51062269	Ensembl:ENSG00000187323
FOS	14	75745531	75748933	Ensembl:ENSG00000170345
GSK3B	3	119540168	119813294	Ensembl:ENSG00000082701
JUN	1	59246463	59249719	Ensembl:ENSG00000177606
KRAS	12	25358180	25403863	Ensembl:ENSG00000133703
LEF1	4	108968704	109090088	Ensembl:ENSG00000138795
MAP2K1	15	66679250	66783882	Ensembl:ENSG00000169032
MAPK1	22	22113946	22221970	Ensembl:ENSG00000100030
MAPK10	4	86931558	87374348	Ensembl:ENSG00000109339
MAPK3	16	30125426	30134541	Ensembl:ENSG00000102882
MAPK8	10	49514720	49647403	Ensembl:ENSG00000107643
MAPK9	5	179660143	179719083	Ensembl:ENSG00000050748
MLH1	3	3703500	37092337	Ensembl:ENSG00000076242
MSH2	2	47630206	47710367	Ensembl:ENSG00000095002
MSH3	5	79950471	80172634	Ensembl:ENSG00000113318

MSH6	2	48010284	48034092	Ensembl:ENSG00000116062
MYC	8	128747680	128755197	Ensembl:ENSG00000136997
PIK3CA	3	178866145	178957881	Ensembl:ENSG00000121879
PIK3CB	3	138371540	138553770	Ensembl:ENSG00000051382
PIK3CD	1	9711789	9789172	Ensembl:ENSG00000171608
PIK3CG	7	106505727	106549425	Ensembl:ENSG00000105851
PIK3R1	5	67511584	67597649	Ensembl:ENSG00000145675
PIK3R2	19	18263973	18281342	Ensembl:ENSG00000105647
PIK3R3	1	46505812	46640573	Ensembl:ENSG00000117461
PIK3R5	17	8782233	8869024	Ensembl:ENSG00000141506
RAC1	7	6414158	6443598	Ensembl:ENSG00000136238
RAC2	22	37621310	37640309	Ensembl:ENSG00000128340
RAC3	17	79989554	79992080	Ensembl:ENSG00000169750
RAF1	3	12625100	12705616	Ensembl:ENSG00000132155
RALGDS	9	135973109	136024597	Ensembl:ENSG00000160271
RHOA	3	49396578	49449409	Ensembl:ENSG00000067560
SMAD2	18	45335328	45457243	Ensembl:ENSG00000175387
SMAD3	15	67357940	67487507	Ensembl:ENSG00000166949
SMAD4	18	48556583	48611412	Ensembl:ENSG00000141646
TCF7	5	133450372	133483901	Ensembl:ENSG00000081059
TCF7L1	2	85360515	85537510	Ensembl:ENSG00000152284
TCF7L2	10	114710006	114927437	Ensembl:ENSG00000148737
TGFB1	19	41836228	41859827	Ensembl:ENSG00000105329
TGFB2	1	218518678	218617961	Ensembl:ENSG00000092969
TGFB3	14	76424440	76449354	Ensembl:ENSG00000119699
TGFBR1	9	101867395	101916474	Ensembl:ENSG00000106799
TGFBR2	3	30647994	30735634	Ensembl:ENSG00000163513
TP53	17	7571739	7590808	Ensembl:ENSG00000141510
