# Supplementary material for: Unraveling the Role of Molecular Profiling in Predicting Treatment Response in Stage III Colorectal Cancer Patients: Insights from the IDEA International Study
Source: Cancers (Basel). 2023 Sep 30;15(19):4819. doi: 10.3390/cancers15194819 (PMC10571744; doi:10.3390/cancers15194819)
Supplement: Supplementary file 1 [file cancers-15-04819-s001.zip › Supplementary Table S3.pdf]

Supplementary Table S3. Signaling pathways and the associated genes for colorectal cancer (CRC) based on Kyoto Encyclopedia of Genes and Genomes (KEGG).

Pathway	KEGG CRC Genes	References [https://www.genome.jp]
Cell cycle	*CCND1, GSK3B, MYC, SMAD2, SMAD3, SMAD4, TGFB1, TGFB2, TGFB3, TP53*	https://www.genome.jp/pathway/hsa04110
p53 signaling pathway	*BAX, BCL2, CASP3, CASP9, CCND1, CYCS, TP53*	https://www.genome.jp/pathway/hsa04115
Apoptosis	*AKT1, AKT2, AKT3, BAD, BAX, BCL2, BIRC5, CASP3, CASP9, CYCS, FOS, JUN, KRAS, MAP2K1, MAPK1, MAPK10, MAPK3, MAPK8, MAPK9, PIK3CA, PIK3CB, PIK3CD, PIK3R1, PIK3R2, PIK3R3, RAF1, TP53, DCC, APPL1*	https://www.genome.jp/pathway/hsa04210
mTOR signaling pathway	*BRAF, GSK3B, KRAS, MAP2K1, MAPK1, PIK3CA, PIK3CB, PIK3CD, PIK3R1, PIK3R2, PIK3R3, RAF1, RHOA*	https://www.genome.jp/pathway/hsa04150
PI3K-Akt signaling pathway	*AKT1, AKT2, AKT3, BAD, BCL2, CASP9, GSK3B, KRAS, MAP2K1, MAPK1, MAPK3, MYC, PIK3CA, PIK3CB, PIK3CD, PIK3CG, PIK3R1, PIK3R2, PIK3R3, PIK3R5, RAC1, RAF1, TP53*	https://www.genome.jp/pathway/hsa04151
Wnt signaling pathway	*APC, APC2, AXIN1, AXIN2, CCND1,CTNNB1, GSK3B, JUN, LEF1, MAPK10, MAPK8, MAPK9, MYC, RAC1, RAC2, RAC3, RHOA, SMAD3, SMAD4, TCF7, TCF7L1, TCF7L2, TP53*	https://www.genome.jp/pathway/hsa04310
TGF-beta signaling pathway	*MAPK1, MAPK3, MYC, RHOA, SMAD2, SMAD3, SMAD4, TGFB1, TGFB2, TGFB3, TGFBR1, TGFBR2,*	https://www.genome.jp/pathway/hsa04350
MAPK signaling pathway	*AKT1, AKT2, AKT3, ARAF, BRAF, CASP3, FOS, JUN, KRAS, LEF1, MAP2K1, MAPK1, MAPK10, MAPK3, MAPK8, MAPK9, MYC, RAC1, RAC2, RAC3, RAF1, TGFB1, TGFB2, TGFB3, TGFBR1, TGFBR2, TP53*	https://www.genome.jp/pathway/hsa04010
ErbB signaling pathway	*AKT1, AKT2, AKT3, ARAF, BAD, BRAF, GSK3B, JUN, KRAS, MAP2K1, MAPK1, MAPK10, MAPK3, MAPK8, MAPK9, MYC, PIK3CA, PIK3CB, PIK3CD, PIK3R1, PIK3R2, PIK3R3, RAF*	https://www.genome.jp/pathway/hsa04012
MSI pathway	*APC, AXIN1, AXIN2, BAD, BAX, BCL2, GSK3B, MLH1, MSH2, MSH3, MSH6, TGFBR2*	https://www.genome.jp/dbget-bin/www_bget?path:map05210
Ras signaling pathway	*AKT1, AKT2, AKT3, BAD, KRAS, MAP2K1, MAPK1, MAPK10, MAPK3, MAPK8, MAPK9, PIK3CA, PIK3CB, PIK3CD, PIK3R1, PIK3R2, PIK3R3, RAC1, RAC2, RAC3, RAF1, RALGDS, RHOA*	https://www.genome.jp/pathway/hsa04014
